# Studies of the dynamics of nuclear clustering in human syncytiotrophoblast

**DOI:** 10.1530/REP-15-0544

**Published:** 2016-06

**Authors:** S J Calvert, M S Longtine, S Cotter, C J P Jones, C P Sibley, J D Aplin, D M Nelson, A E P Heazell

**Affiliations:** ^1^ Maternal and Fetal Health Research CentreInstitute of Human Development, School of Medicine, University of Manchester, Manchester, UK; ^2^ St Mary’s HospitalCentral Manchester University Hospitals NHS Foundation Trust, Manchester Academic Health Science Centre, Manchester, UK; ^3^ Department of Obstetrics and GynecologyWashington University School of Medicine, St Louis, Missouri, USA; ^4^ School of MathematicsAlan Turing Building, University of Manchester, Manchester, UK

## Abstract

Syncytial nuclear aggregates (SNAs), clusters of nuclei in the syncytiotrophoblast of the human placenta, are increased as gestation advances and in pregnancy pathologies. The origins of increased SNAs are unclear; however, a better appreciation of the mechanism may give insight into placental ageing and factors underpinning dysfunction. We developed three models to investigate whether SNA formation results from a dynamic process of nuclear movement and to generate alternative hypotheses. SNA count and size were measured in placental explants cultured over 16 days and particles released into culture medium were quantified. Primary trophoblasts were cultured for 6 days. Explants and trophoblasts were cultured with and without cytoskeletal inhibitors. An *in silico* model was developed to examine the effects of modulating nuclear behaviour on clustering. In explants, neither median SNA number (108 SNA/mm^2^ villous area) nor size (283 μm^2^) changed over time. Subcellular particles from conditioned culture medium showed a wide range of sizes that overlapped with those of SNAs. Nuclei in primary trophoblasts did not change position relative to other nuclei; apparent movement was associated with positional changes of the syncytial cell membrane. In both models, SNAs and nuclear clusters were stable despite pharmacological disruption of cytoskeletal activity. *In silico*, increased nuclear movement, adhesiveness and sites of cytotrophoblast fusion were related to nuclear clustering. The prominence of SNAs in pregnancy disorders may not result from an active process involving cytoskeleton-mediated rearrangement of syncytial nuclei. Further insights into the mechanism(s) of SNA formation will aid understanding of their increased presence in pregnancy pathologies.

## Introduction

The placenta is a transient organ, the correct development of which is essential for a healthy pregnancy. In the human placenta, the maternal surface of placental villi is covered by syncytiotrophoblast that is in direct contact with maternal blood. This essential cell layer performs many functions including gas exchange, hormone production, immune protection and the transport of nutrients from mother to fetus (Boyd & Hamilton 1970). To allow growth of the placenta, subjacent mononucleate progenitor cells, cytotrophoblasts, replicate and fuse into the terminally differentiated syncytiotrophoblast (Boyd & Hamilton 1967). Pregnancy disorders such as pre-eclampsia are characterised by abnormal placental development, alterations in trophoblast apoptosis and release of trophoblast-derived fragments into the maternal circulation (Chaddha et al. 2004, Hahn et al. 2005, Goswami et al. 2006, Heazell et al. 2008a).

In the syncytiotrophoblast, nuclei can cluster to form syncytial nuclear aggregates (SNAs). *In vivo*, SNAs accumulate throughout pregnancy; they are especially noted in histological analyses of prolonged pregnancies (post-term, >40 weeks; Jones & Fox 1978) but are found at earlier gestational ages and seen in greater abundance in pregnancies complicated by pre-eclampsia (Tenney & Parker 1940, Al-Allaf et al. 2008, Corrêa et al. 2008, Calvert et al. 2013). Similarly, *in vitro* formation of SNAs is increased by oxidative stress (Heazell et al. 2007), and culture of isolated trophoblast cells results in spontaneous fusion between 24 and 48h (Kliman et al. 1986), with the possibility that the nuclei will cluster, here termed as syncytial nuclear clusters (SNCs).

Syncytial knots, a subtype of SNA, form more towards term and in the past have often been thought to represent ‘ageing’ of the placenta. In contrast to syncytial knots, other SNA subtypes, and more specifically syncytial sprouts, may reflect placental growth (Cantle et al. 1987, Burton & Jones 2009). The morphology of syncytial knots shows nuclei with dense heterochromatin. This nuclear condensation was previously thought to indicate a trajectory towards apoptosis (Huppertz et al. 2006); however, we previously characterised SNAs in normal-term placentas and demonstrated that most of the constituent nuclei are not apoptotic, although knots are more likely than other types of SNA to be apoptotic (Coleman et al. 2013). Similarly, others have found little evidence that there are apoptotic changes in normal syncytiotrophoblast (Burton & Jones 2009, Longtine et al. 2012a). Instead, SNAs, and particularly syncytial knots, have been found to show epigenetic changes associated with oxidative damage that could lead to heterochromatin formation (Fogarty et al. 2013), without necessary progression to apoptosis or shedding of apoptotic debris.

Despite the long-standing association between SNAs and pregnancy pathologies, unanswered questions remain. In a recent review, Mayhew (2014) proposed avenues for further investigation of SNAs such as understanding why SNAs, including knots, form. Another avenue was to determine the benefits of allowing oxidative-damaged nuclei with condensed chromatin to accumulate, if SNAs are not preferentially extruded. Mayhew (2014) also raises questions about the relevance of increased density of SNAs to pre-eclampsia, particularly an increase in syncytial knots (Calvert et al. 2013). The mechanism of SNA formation remains unknown (Aplin 2010); however, cytoskeletal proteins are found in association with them (Jones & Fox 1977, Coleman et al. 2013). As actin microfilaments and microtubules are involved in nuclear movement and anchorage in syncytia of skeletal muscle, *Danio rerio* and *Caenorhabditis elegans* development (Malone et al. 1999, Frock *et al.* 2006, Carvalho et al. 2009), we hypothesised that they could be involved in SNA formation in human syncytiotrophoblast.

Our objectives for this study were to (1) use placental villous explant cultures to examine the dynamics of SNAs; (2) use primary trophoblast cells *in vitro* to observe the formation of syncytial nuclear clusters (SNCs, the form we identified SNAs take in cell culture); (3) explore cytoskeletal disruption in these models to see whether this affects SNA or SNC numbers, giving insight into whether SNAs are formed or held together using dynamic cytoskeletal rearrangements, and (4) use data obtained from these *in vitro* models to develop an *in silico* model of nuclear clustering to explore factors that may influence the formation and maintenance of SNAs or SNCs. To address objectives 1 and 2, this study extended the length of *in vitro* culture from that typically employed, as estimates suggest that *de novo* synthesis of SNAs could take weeks (Huppertz et al. 2002, 2003) and that SNC formation would occur in more mature syncytia. Consequently, an assessment of viability was conducted before experiments to disrupt the cytoskeleton. The effect of pharmacological agents was examined in the cultured trophoblast model at two time points: (i) after SNCs were considered to have formed at 72h and (ii) during syncytialisation at 40–42h. In placental explants, it was anticipated that SNAs would develop from existing nuclei during culture; therefore, pharmacological agents were added after 24h, as previous experiments altering culture conditions at this time had an effect on SNAs (Heazell et al. 2007).

## Materials and methods

### Placental collection, tissue and cell culture

All reagents were purchased from Sigma-Aldrich (Poole, UK, for explant work and St Louis, USA, for cell preparations) unless otherwise stated. Placentas used for explant work were obtained under tissue biobank ethics from St Mary’s Hospital Maternity Unit (Manchester, UK) following informed consent, approved by North West (Haydock Park) Research Ethics Committee (Ref: 08/H1010/55). Placentas were selected if delivered after 37 weeks of gestation and with no maternal or foetal morbidities during pregnancy (demographic information in Supplementary Table 1, see section on supplementary data given at the end of this article). Tissue processing was started within 30min of delivery; explants were made from three randomly selected areas of the placenta and cultured in medium using Netwells at the medium/gaseous interface, as described previously (Siman et al. 2001). CMRL-1066 culture medium was supplemented with 10% fetal bovine serum, NaHCO_3_ (2.2mg/mL), penicillin G (100IU/mL), streptomycin sulphate (100μg/mL), l-glutamine (100μg/mL), retinol acetate (1μg/mL), insulin (1μg/mL) and hyaluronic acid (1μg/mL) (pH 7.2; Invitrogen, Life Technologies). Villous explants were cultured for up to 16 days, which was considered sufficient to enable the kinetics of aggregation and shedding to be observed as it has been hypothesised that SNAs form and are shed within 14–28 days (Huppertz et al. 2002, 2003). Normoxia for term placenta has been estimated to be between 6 and 13% oxygen (O_2_) tension (Jauniaux et al. 2000, Sullivan et al. 2006, Heazell et al. 2008b, Pringle et al. 2010); however, cultured cells may take up gases more quickly than the gases can diffuse, meaning that they are usually hypoxic (Metzen et al. 1995, Pettersen et al. 2005, Tuuli et al. 2011, Chen et al. 2013). Therefore, it was decided to culture explants at both 6% O_2_ with 5% CO_2_ and 89% N_2_ and 20% O_2_ with 5% CO_2_ and 75% N_2_. Explants were weighed and fixed for 24h in 10% neutral buffered formalin from fresh tissue and at day 4, 8, 12 and 16 (*n*=6). Medium was changed daily, with conditioned medium collected and stored at −80°C.

For experiments with purified primary trophoblasts, placentas were collected under informed consent, approved by the Institutional Review Board of Washington University School of Medicine in St Louis, MO, USA. Normal-term placentas of 38–40 weeks of gestation (*n*=3) were obtained after uncomplicated Caesarean section. Primary trophoblast cells were isolated as described by Chen et al. (2006) and plated at a density of 200,000 cells/cm^2^ to encourage an even, single layer for best visibility. Trophoblasts were cultured in a 5% CO_2_/air environment at 37°C in DMEM supplemented with 10% foetal bovine serum (Invitrogen, Life Technologies), 20mM HEPES pH 7.4 (Sigma), 100units/mL penicillin and 100μg/mL streptomycin, for the times indicated, with daily changes of medium. As noted, selected experiments also received 100ng/mL epidermal growth factor (EGF; Millipore) added to cultures that have been suggested to increase the rate and extent of syncytiotrophoblast formation *in vitro* (Morrish et al. 1987, 1997, Johnstone et al. 2005) and to reduce trophoblast stress-induced apoptosis (Garcia-Lloret et al. 1996, Moll et al. 2007, Humphrey et al. 2008).

### Analysis of tissue viability and hormone release from syncytiotrophoblast

Tissue viability was assessed by lactate dehydrogenase (LDH) release using a cytotoxicity detection kit (Roche Applied Science) and production of the hormones human chorionic gonadotropin (hCG) and human placental lactogen (hPL) into conditioned culture medium using kits hCG ELISA EIA-1469 (DRG International, Springfield, NJ, USA) and hPL ELISA EIA-1283 (DRG International) (Audette et al. 2010). Proliferation and apoptosis were measured as described previously (Heazell et al. 2008b). 

### Inhibition of intracellular motility

Explants (*n*=6) were cultured in 20% O_2_ and treated after 24h with the following cytoskeletal disruptors (all from Sigma): cytochalasin D (actin polymerisation inhibitor), nocodazole (microtubule polymerisation inhibitor), paclitaxel (microtubule stabiliser) at 0.1, 1 or 10mM or with solvent control (0.2% dimethyl sulfoxide (DMSO)) for 20h before washing and culturing the explants for a further 48h; treated explants were weighed and fixed at day 4.

To assess SNC stability in cells, primary trophoblasts were cultured with EGF for 72h and then treated for 6h with either 10μM nocodazole, 1μM cytochalasin D or both nocodazole and cytochalasin D. Control cultures were treated with 0.2% DMSO. Additional experiments were conducted to examine whether SNC formation was inhibited by culturing primary trophoblasts for 40–42h before 18h of treatment with drugs or control at the same concentrations as the other trophoblast experiments. After cytoskeletal disruptor treatments, primary trophoblasts were fixed for imaging.

### Examination of shed particles

Explant-conditioned culture medium was collected at intervals of 48h for 16 days and processed immediately (*n*=4). Medium was centrifuged at 9000 ***g*** for 4min using MiniSpin (Eppendorf, UK). The pellet was resuspended in 200μL phosphate-buffered saline (PBS) and stained with 4′,6-diamidino-2-phenylindole (DAPI) and CellTracker Orange (Invitrogen, Life Technologies). Briefly, 0.1μL CellTracker Orange and 0.5μL DAPI were added for 10min at room temperature to stain all nuclei and cytoplasm, followed by centrifugation at 900 ***g*** for 5min. The pellet was resuspended away from light, washed for 3min and centrifuged at 900 ***g*** for 5 min. The final pellet was resuspended in 100μL PBS and placed in a 96-well dish. Particle size and number were analysed using the BD Pathway Bioimager 855 High Content Screening System (BD Bioscience, San Jose, CA, USA) and Image J 1.45s (NIH, available at http://rsb.info.nih.gov/nih-image/) (Schneider et al. 2012). Particles from frozen conditioned explant media were also collected by centrifugation onto 3-aminopropyltriethoxysilane (APES)-coated slides using Shandon Cytofunnel EZ singles with CytoSpin 4 Cytocentrifuge (Thermo Scientific). The particles were stained with haematoxylin and eosin and imaged (*n*=3). A threshold of 80μm^2^ was employed as this was estimated to be the largest size for red blood cells and single trophoblast cells and <0.2% of SNAs in fresh tissue were smaller than 80μm^2^.

### Histological examination

Fixed explants were wax embedded and 5μm sections were mounted onto APES-coated slides. Cells were fixed by a 20-min exposure to ice-cold methanol.

### Quantification of SNA number and size

Sections were stained with haematoxylin and eosin to assess SNA number and size. SNAs were defined as clusters of ten or more nuclei protruding slightly from the villus edge, from either one villus or linking two villi (Cantle et al. 1987). Ten fields of view were imaged and SNAs were counted and their area measured. Images were analysed using an Olympus BX41 microscope with ImageProPlus 7.0 software (Media Cybernetics, Rockville, MD, USA).

### Immunohistochemistry

Endogenous peroxidase activity was quenched using 3% aqueous hydrogen peroxide and non-specific interactions blocked with 10% animal serum. Sections were incubated with 1.1μg/mL mouse monoclonal M30 Cytodeath antibody (Roche), 0.16μg/mL mouse monoclonal Ki67 antibody (Dako MIB-1 clone) or non-specific mouse IgG-negative control (1.1 or 0.16μg/mL as appropriate). Biotinylated goat anti-mouse (Dako; 1:200) and avidin–peroxidase (5μg/mL in 0.125M TBS plus 0.347M NaCl) (Jones et al. 1987) were applied and a 3,3-diaminobenzidine treatment was performed to visualise staining. Nuclei were counterstained with Harris’ haematoxylin. Ten fields of view were imaged as above and analysed for positive trophoblast nuclei as a percentage of total placental nuclei. Only Ki67-positive cytotrophoblasts were counted as assessed by proximity to the syncytiotrophoblast; other positive nuclei were not included. M30-neoepitope staining was measured in all positive areas as a total of whole explant area.

### Immunofluorescence

Immunofluorescence was performed on sections of explant tissue as described previously (Coleman et al. 2013). Briefly, mouse monoclonal anti-β actin AC-74 (Sigma, 1.25μg/mL), anti-γ actin 2-2.1.14.17 (Sigma, 4μg/mL), anti-α tubulin DM1A (1μg/mL; Abcam), anti-β tubulin (0.46μg/mL; Sigma SAP.4G5), anti-cytokeratin 7 (clone OV/TL 12/30, 4.6μg/mL; Dako) or corresponding concentrations of non-immune isotype-matched mouse IgG were incubated on sections followed by incubation with rabbit anti-mouse FITC (1:200; Dako) and mounted with Vectashield with DAPI or PI to counterstain nuclei (Vector, Burlingame, CA, USA). A Zeiss AxioObserver inverted microscope (Carl Zeiss) was used to visualise staining and AxioVision Rel. 4.8 was used to analyse images.

For cell immunofluorescence, a 1% bovine serum albumin (BSA) block was used. Primary antibodies were 1.25μg/mL mouse anti-E-cadherin 610181 (BD Bioscience, San Jose, CA, USA), 5μg/mL rabbit anti-E-cadherin 40772 (Abcam), 2μg/mL mouse anti-α tubulin 7291 (Abcam) or 4.4μg/mL β-actin A2228 (Sigma) and secondary antibodies, used at 10μg/mL, were Alexa Fluor anti-mouse 488, A11029; Alexa Fluor anti-rabbit 488, A11034; or Alexa Fluor anti-mouse 546, A11003 (all Invitrogen, Life Technologies). After staining nuclei with 5μM DRAQ5 (Biostatus Limited, Leicestershire, UK) and mounting using Fluoro-Gel (Electron Microscopy Sciences, Hatfield, PA, USA), images were acquired using a Nikon ECLIPSE E800 (Nikon) or Olympus FV-500 microscope system equipped with a 60× oil immersion lens, confocal laser scanning head and three lasers with emissions of 488, 546 and 633nm. For each cell preparation, 12 fields of view were selected randomly and captured images were analysed using Image J 1.45s (NIH, available at http://rsb.info.nih.gov/nih-image/).

### Measurement of internuclear distance

In cultured trophoblasts, our analyses of internuclear distance were restricted to ‘large syncytia’ (those with 6 or more nuclei) based on previous work which found many syncytia in cultured trophoblasts contain three to five nuclei, with syncytia with 6 or more nuclei representing ∼30% of the total (Frendo et al. 2003). We chose these large syncytia for analysis, as they provide more area with a greater ability to detect non-random nuclear localisation. Nuclear positioning was quantified only in trophoblasts and syncytia with clearly defined E-cadherin staining that allowed us to clearly identify the cell boundaries. Measurements for cytotrophoblasts were taken from unfused cells with at least one border, estimated as at least half the cell membrane outline, in contact with other unfused cytotrophoblasts, as determined by E-cadherin staining. These criteria were chosen for cytotrophoblast measurements in order to make comparisons with syncytialised nuclei, which lie next to each other. Internuclear distance was determined by measuring the distance between the edge of a nucleus and the edge of its nearest neighbouring nucleus. The designation of SNCs used was based on the results of the study reported here, with a cluster defined as at least six nuclei, all with nearest neighbour internuclear distances of ≤3μm; nuclei not meeting this definition were identified as not residing in a cluster. Cells in culture with a highly condensed nuclear morphology are likely to have undergone apoptosis and be non-viable (Longtine et al. 2012b) and were excluded from the analysis.

### Measurement of cytoplasmic area per nucleus

Cytoplasmic area per nucleus was determined by measuring the cytoplasmic area of adjacent cytotrophoblasts or ‘large syncytia’ using Image J 1.45s (NIH, available at http://rsb.info.nih.gov/nih-image/) to outline the E-cadherin-defined cell borders and dividing that area by the number of nuclei counted in that group of cytotrophoblasts or that syncytium.

### Time-lapse microscopy

Time-lapse microscopy was performed using an inverted Nikon TE2000-U microscope. Cells were incubated in a humidified chamber at 37.0°C with 5% CO_2_ and 20% O_2_ and phase–contrast images were recorded every 5 or 10 min, typically for 18h, as noted in the figure legends. Videos were generated using Image J 1.45s (NIH, available at http://rsb.info.nih.gov/nih-image/) (Schneider et al. 2012) and annotated in Blender 2.49 (Blender Foundation, Amsterdam, The Netherlands).

### Statistical analysis

Statistical signiﬁcance was assessed using Graphpad Prism (Version 5.03). Data were analysed using Kruskal–Wallis test with Dunn’s *post hoc* test or, when comparing two data sets, using two-way ANOVA. *P* values ≤0.05 were deemed significant.

### In silico model

We modelled the movement of the nuclei as a set of interacting Brownian motions in a two-dimensional cross section of the syncytiotrophoblast layer. Within this two-dimensional approximation, we were able to include the important features that we hypothesise to play a major role in the formation of large clusters of nuclei within the syncytium. On a long timescale, the nuclei diffuse within the syncytium. The contact forces between cell membranes and the nuclei (and adhesive forces for internuclear interactions) are modelled using a potential that models the forces that each nucleus is subjected to as time evolves. If the radius (*R*) of a nucleus overlaps with that of another, or with the cell membrane, a large repulsive force is exerted. If the perimeters of two nuclei are within a distance *R*<< 1 of each other, then there is a smaller adhesive force that tends to keep the nuclei close to one another.

To include random variation in the thickness of the syncytium, the upper boundary was produced using a polynomial interpolation of a subsampled Ornstein–Uhlenbeck process, leading to a smooth mean zero function (Uhlenbeck & Ornstein 1930). An Ornstein–Uhlenbeck process has a Gaussian stationary distribution, and we picked the standard deviation in such a way that the variation is less than 0.75 nuclear diameters with 99.7% confidence. The amplitude of this variation is altered in one set of experiments, in which the variation is multiplied by the parameter A. The mean thickness of the cell layer was chosen to be 1.5 nuclear diameters, the parameter that we used to scale the space. The lower boundary was kept as a straight line for simplicity. The length of the domain was chosen to be 250 nuclear diameters, so that using rough estimates of nuclear density at term (29.4% of the volume (Mayhew et al. 1999)), there would be 140 nuclei in the modelled syncytial area. This length is sufficiently long that the boundary effects on the result due to this truncation would be minimal.

The contact forces (repulsion and adhesion) between each pair of nuclei were modelled through a potential function *V*. If the distance between the centres of two nuclei is less than 1 nuclear diameter, then they are overlapping, and the potential function exerts a strong repulsive force. If the two nuclear centres are within a distance x of each other, where 1<×<1+*R*, and *R*=0.05 (units are expressed as nuclear diameters), then they are assumed to be ‘stuck together’, and a smaller force is exerted on the two nuclei towards each other. The size of this force is determined by a “stickiness”, or adhesive, parameter *S*. If the centres of the two nuclei are farther than 1+R away from each other, then it is assumed that there is no interaction between them. The potential function used as default within the model is shown in Supplementary Fig. 1, and the parameters from the equation with the values they hold are listed in Supplementary Table 2.

In addition to these forces, each nucleus is also subject to a slow scale diffusion in two dimensions, with diffusion constant D. The additional parameter for preferential sites of fusion is *σ*, the units of which are expressed as nuclear lengths. The distribution of the fusion sites in this experiment is given by a normal distribution with mean L/2 and variance *σ*^2^.

Using this model, we investigated factors that could cause nuclei to form SNAs. For the *in silico* model, any clustering of nuclei was measured, thus ≥2 nuclei adhered to one another were considered a cluster. We explored four different scenarios to give insight into what causes changes in the cluster size and distribution: (i) the adhesion of nuclei in internuclear interactions, (ii) the rate of diffusion of the nuclei within the syncytium, (iii) preferential sites for the fusing of cytotrophoblast nuclei into the syncytium and (iv) changing/narrowing the width of the cell during pregnancy. As the model is stochastic, there is random variation in the results, with each run of the model producing a different configuration of clusters. Therefore, to see the effect on the cluster size distribution, each scenario was repeated 500 times. A scenario is a particular set of values of the parameters. Mostly only one parameter was varied each time; however, as diffusion of the nuclei has a negative correlation and nuclear adhesion a positive correlation on clustering, it was necessary to change both these values in one experiment (model ii) to maintain the adhesiveness at the overall same value, while exploring the effect of increased diffusion. At the end point, any two nuclei were considered to be ‘connected’ if they were within a distance 1+*R* of each other, i.e. within the radius of interaction. A matrix is formed, each entry of which tells us whether each pair of nuclei is connected. From this, using an implementation of Tarjan’s algorithm (Tarjan 1972), all the clusters were identified.

## Results

**Figure 1:**
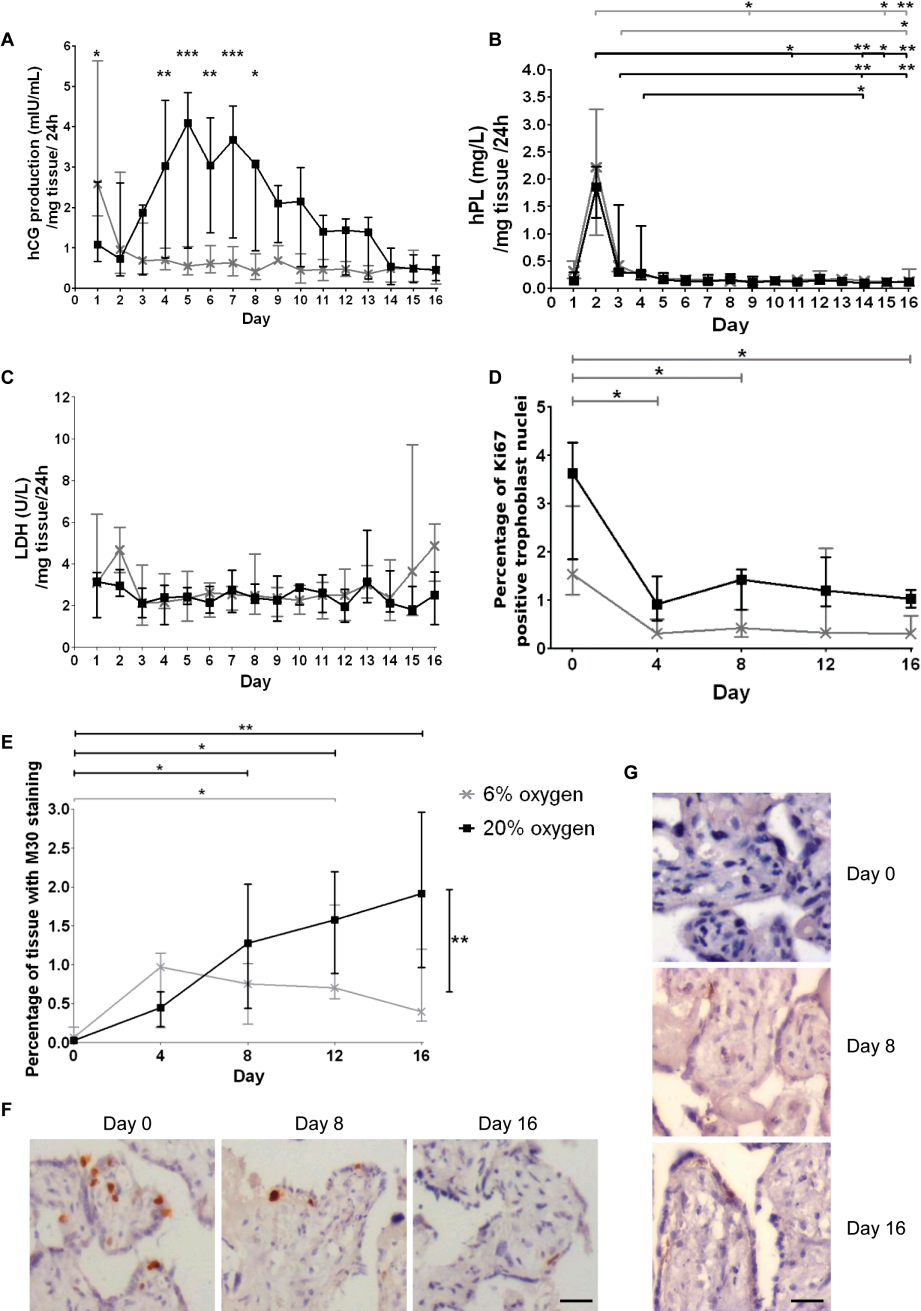
(A) There was a significant difference in hCG production between the two oxygen concentrations as assessed by two-way ANOVA at days 1 and 4–8. (B) A black colour significance bar at the top (20% O_2_) or grey colour significance bar at the top (6% O_2_) relates to a significant difference between hPL production between days 2, 3 or 4 and a later time point assessed by Kruskal–Wallis test. (C) There were no significant differences in LDH release across the culture period. (D) Ki67 staining reduced from day 0 compared with days 4, 8 and 16 at 6% O_2_. (E) Apoptosis, assessed by staining for the M30 neoepitope, significantly increased at day 16 in 20% O_2_ compared with 6% O_2_ (black colour significance bar at right) (two-way ANOVA). Staining was increased after day 8 in 20% O_2_ (black colour significance bars at top) and between days 0 and 12 in 6% O_2_ (grey colour significance bar at top) Kruskal–Wallis test. (F) Representative images of the reduction in Ki67-positive trophoblasts across the time frame and (G) M30 neoepitope increased staining with time at 20% O_2_. Scale bars, 20 μm; *P<0.05, **P<0.01, ***P<0.001 (*n*=6; median and interquartile range). The key next to graph 1E applies to all graphs in the figure.

Before examining SNA dynamics in the placental explant model, we characterised nuclear distribution over time and in response to altered oxygenation to determine optimal conditions for extended explant culture. At 20% O_2_, hCG was continuously released, with peak levels between day 4 and 8. At 6% O_2_, release of hCG occurred on day 1 after which lower baseline levels of hCG were observed (Fig. 1A). The pattern of hPL release in cultured explants was similar at both 6 and 20% O_2_, with a rise at day 2, followed by consistent, low secretion for the remaining 16 days of culture (Fig. 1B). There was no significant increase in LDH in the conditioned culture medium at either oxygen concentration, indicating that there is no increase in the number of necrotic cell death (Fig. 1C). Cytotrophoblasts remaining in cycle, as assayed by Ki67 staining, were reduced compared with fresh tissue; however, the level was maintained throughout culture (Fig. 1D and F). Cleaved cytokeratin 18 staining increased from very low levels (<0.5%) in fresh tissue to approximately 2% of explant area by day 16 of culture in 20% O_2_ (Fig. 1E and G), whereas at 6% O_2_, this marker only reached approximately 1% of explant area; although there was a significant increase in staining between fresh tissue and day 12, the statistical significance was lost at day 16.

**Figure 2:**
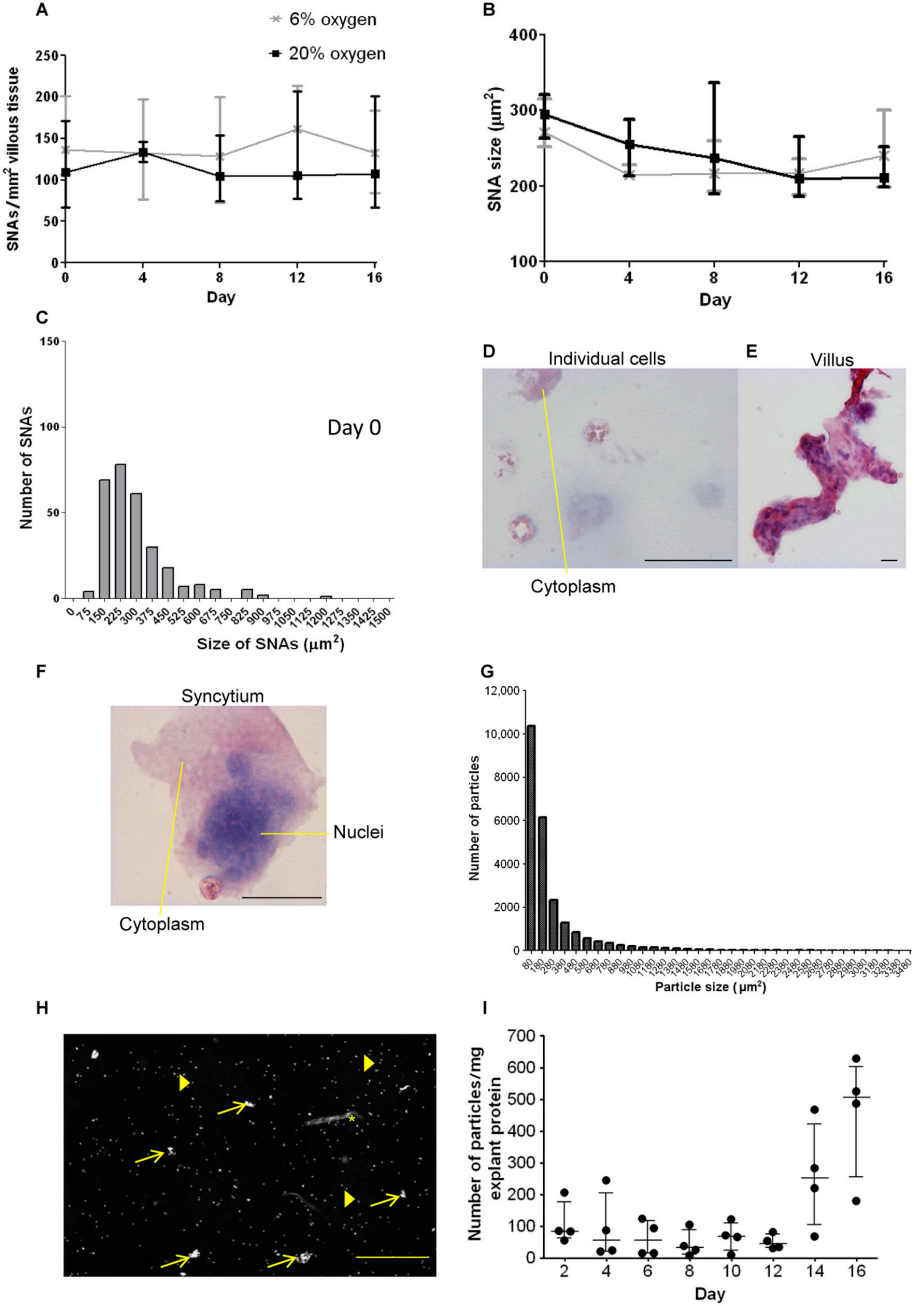
(A) The density and (B) size of SNAs do not change over the 16 days culture period or in different oxygen tensions when assessed by two-way ANOVA and Kruskal-Wallis test (*n* = 6; median and interquartile range). (C) The size range of SNAs demonstrates that the majority are between 150 and 375 μm (*n* = 11; fresh tissue), one point of 1826 was excluded from this graph. Tissue fragments collected from explant-conditioned medium stained with haematoxylin and eosin, (D) individual cells, (E) villous tissue lost from an explant and (F) structure similar to an SNA with several nuclei grouped closely together (scale bars = 20 μm). (G) Most particles analysed had a measurement in the lower (SNA) size range (*n* = 4). (H) A representative image of DAPI-stained particles shed into culture media; approximately 1/10 of the well is shown. Examples of single cells (filled arrows) are shown that were below 80 μm; several particles that could be SNAs are indicated by open arrows. One villus fragment is marked by asterisk “*”. Scale bar = 200 μm. (I) There was no significant change in the number of fragments ≥80 μm^2^ lost over time into culture medium (*n* = 4; Kruskal-Wallis test).

We next combined histological examination of nuclear distribution with time at various oxygen levels, with cytological evaluation of shed material. When explants were assessed every 4 days up to 16 days, no significant change in SNA density or size was observed. There were no differences in these parameters between the 6 and 20% O_2_ conditions (Fig. 2A and B). In fresh tissue, the range of SNA sizes was 80–900μm^2^ with four exceptions, and over 80% of SNAs were within 80–375μm^2^ (Fig. 2C). Shed material included particles ranging from single cells to pieces of villous tissue and detached syncytiotrophoblast that may contain SNAs (exemplified in Fig. 2D, E and F). The shed particle size distribution overlapped with the range observed in SNAs from tissue sections but extended to some much larger particles (approximately three times the size of the largest SNAs) and included some villous fragments already known to detach in the explant model (Fig. 2G). Examples of images taken highlight many single cells, particles that could be SNAs, and one particle that has a villous morphology (Fig. 2H). There was no significant change in particles shed per mg of explant protein over time (Fig. 2I; Kruskal–Wallis) with a range of 11–636 particles per 48h per mg explant protein.

Histological analysis of the explants revealed some degree of syncytiotrophoblast shedding from day 4, with regrowth of the syncytiotrophoblast noticeable from day 8, as described previously (Siman et al. 2001). Newly differentiated nuclei were most obviously seen on day 8 and 12, and although they were often adjacent to one another, the nuclei present seemed less numerous and the regrown syncytiotrophoblast was not seen to host SNAs. In placental explants, SNAs were associated with intermediate filament proteins consistently throughout the culture period; in particular, strong cytokeratin 7 immunoreactivity surrounded SNAs in fresh tissue up to day 8 (Supplementary Fig. 2). Tubulin staining was found in close proximity to SNAs in fresh tissue up to day 8; however, there was limited staining on day 12 and 16. β-Actin was easily observed in fresh tissue but was harder to identify in the syncytiotrophoblast after that time point, although staining was visible within fibrin deposits (Supplementary Fig. 3).

**Figure 3:**
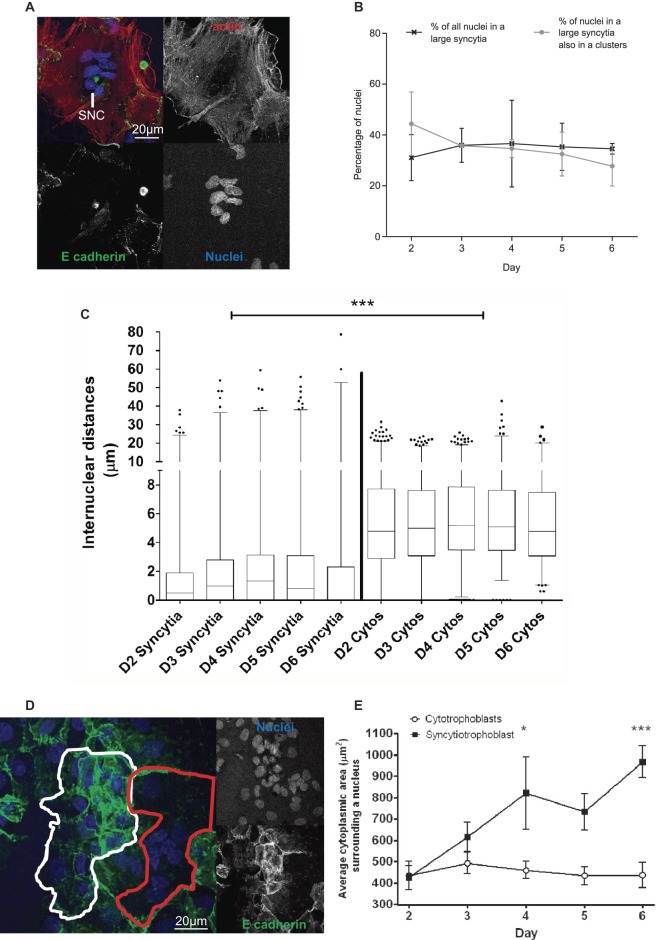
(A) Representative image of an SNC in isolated cytotrophoblast cell culture. (B) There was no change in the percentage of nuclei in large syncytia or in SNC over 6 days of culture. (C) Internuclear distances were smaller in large syncytia compared with cytotrophoblast cells (median and interquartile range in box plot with whiskers extending between the 1st and 99th percentile; *** *P* < 0.001). (D) An example of areas selected for measurements as ‘adjacent cytotrophoblast cells’ and large syncytia are shown in white and red colour respectively. (E) Large syncytia had a significantly greater ratio of cytoplasmic area to nuclei than cytotrophoblasts on days 4 and 6 of culture. Graph shows median and interquartile range assessed by Kruskal-Wallis test; *P < 0.05, ***P < 0.001 (*n* = 3).

In primary trophoblast culture, cells were plated as mononucleate cytotrophoblasts and progressively fused, ultimately resulting in most nuclei being within syncytia (cells with two or more nuclei/plasma membrane boundaries). Groups of associated nuclei, similar to SNAs, were apparent after 2 days of culture. Here, we refer to these as “syncytial nuclear clusters” (SNCs) (Fig. 3A). The proportion of nuclei in “large syncytia” (>6 nuclei) (∼40%) showed no significant change over 2–6 days in culture. Similarly, the proportion of nuclei in these “large syncytia” that had gathered into SNCs showed no significant change over the culture period (Fig. 3B). Trophoblasts and syncytia that had undergone apoptosis as indicated by condensed nuclei visible in phase–contrast microscopy (Longtine et al. 2012b) were visible in culture from day 3. Qualitatively, these apoptotic regions were sparse and covered small areas at day 3, becoming increasingly common by days 5–6 (Supplementary Fig. 4). For subsequent analyses, measurements were only taken from trophoblasts without condensed nuclei. The median internuclear distance was significantly smaller in syncytia than in unfused adjacent cytotrophoblasts: 0.81μm vs 4.99μm (*P* > 0.001); however, neither value changed significantly during culture (Fig. 3C). Although internuclear distance showed little change, there was a significantly higher cytoplasmic area per nucleus in syncytia (Fig. 3D) after 4 days (*P*≥0.05) and 6 days of culture (*P*≥0.001) in comparison to that seen in unfused cytotrophoblast cells, which retained a similar cytoplasmic area per nucleus throughout the culture period (Fig. 3E).

**Figure 4:**
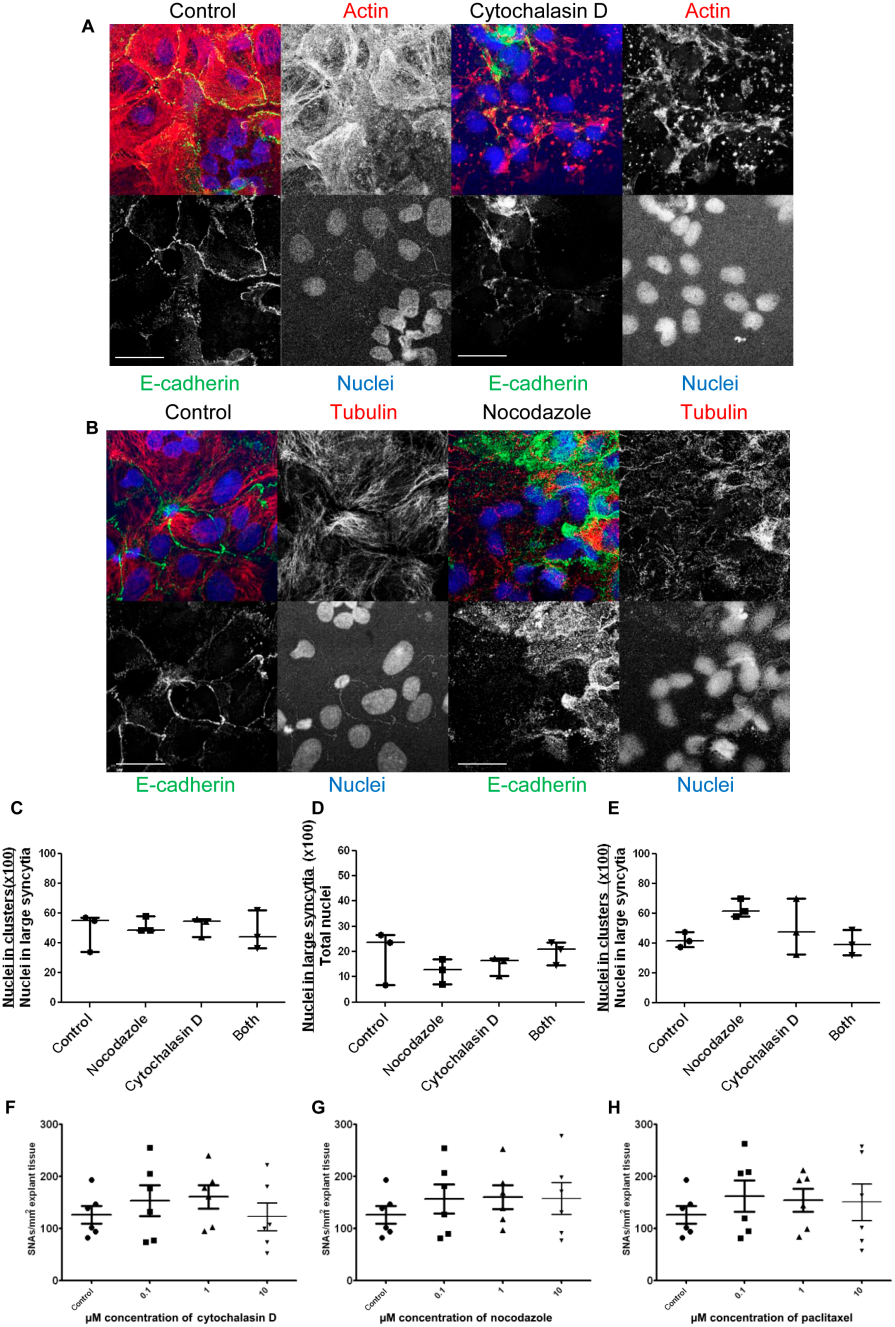
Representative images of (A) control cells (red gain 7.60) and cells treated with cytochalasin D (red gain 7.45) and (B) control cells (red gain 7.60) and cells treated with nocodazole (red gain 8.05) for 2 h. Scale bar=20 μm. Gains were changed here, only, to show more clearly the differences in the organisation of the cytoskeleton. The higher gain needed for signal (B) and disorganised structure of the cytoskeletal proteins (A and B) demonstrate successful disruption of actin and tubulin respectively. (C) Treatment with cytochalasin D and nocodazole at 72 h did not change the percentage of nuclei in SNCs. (D) Addition of cytochalasin D and nocodazole at 40–42 h did not change the number of cells syncytialising or (E) the percentage of nuclei in SNCs (*n*=3). There was also no significant change in the number of SNAs in placental explants after treatment with 0.1 μM, 1 μM or 10 μM, (F) cytochalasin D, (G) nocodazole or (H) paclitaxel (*n*=6) (Kruskal–Wallis test).

To investigate whether cytoskeletal components were involved in SNC formation and maintenance, microfilaments and microtubules were disrupted in primary cell culture by exposure to cytochalasin D or nocodazole respectively. Based on immunofluorescence images, E-cadherin continued to identify cell boundaries after 2h of cytochalasin D and nocodazole treatment; however, the treatments disrupted actin and tubulin respectively (Fig. 4A and B). Cytochalasin D treatment changed filamentous actin to a globular form and nocodazole treatment changed longer organised microtubules, particularly seen around the edges of syncytia, to disrupted shorter fragments. Treatment with cytochalasin D or nocodazole for 6h did not significantly diminish the proportion of nuclei in SNCs in syncytiotrophoblast (Fig. 4C). Similarly, treatment with cytochalasin D or nocodazole for 18h before fusion had reached a maximum level at 40–42h of culture had no effect on the percentage of nuclei in “large syncytia” (>6 nuclei) or in SNCs (Fig. 4D and E respectively). Actin was depolymerised in explants cultured for 24h and then treated with cytochalasin D for 20h (Supplementary Fig. 5). Nocodazole depolymerised tubulin in explants and paclitaxel stabilised tubulin in explants over the same time frame (Supplementary Fig. 5). However, there was no significant change in SNA density after treating explants with cytochalasin D (Fig. 4F), nocodazole (Fig. 4G) or paclitaxel (Fig. 4H).

Time-lapse imaging of cultured primary trophoblasts revealed that nuclei in SNCs were remarkably stable in their position within the cell and in their positions relative to one another. Nuclear movement was only observed with associated movement of the cell membranes, for instance, during the initial spreading of trophoblasts on the tissue culture plate (Supplementary Video 1). Between day 2 and 4, there was little change in relative nuclear positions (Supplementary Videos 2 and 3). Cytochalasin D and nocodazole affected the ability of the cultured trophoblasts and syncytia to maintain their shape; again cell membrane movement was associated with nuclear movement (Supplementary Videos 4, 5 and 6).

**Figure 5:**
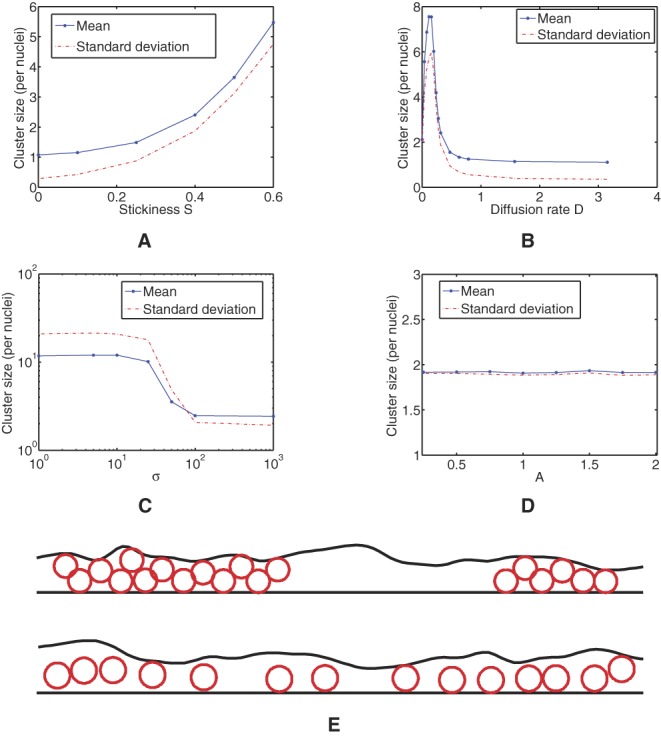
(A) The average size of cluster of which a given nucleus is likely to be a member, as a function of the stickiness parameter S. (B) The average size of cluster of which a given nucleus is likely to be a member, as a function of the diffusion parameter D. (C) The average size of cluster of which a given nucleus is a member, as the preferential site parameter *σ* is altered. The distribution of the fusion sites in this experiment is given by a normal distribution with mean L/2 and variance *σ*^2^. (D) The average size of cluster of which a given nucleus is a member, as the amplitude A is altered. (E) Visualisations of a section of the syncytium with two clusters of nuclei (top) and a section with only small clusters (bottom), computed using our model.

A mathematical model was devised as a tool for examining the effects of factors that might affect nuclear distribution, assuming stochastic progression from a baseline. The set variables included diameter that cytotrophoblast fusion can occur within, tendency of nuclei to remain associated once in proximity to one another, rate of nuclear movement within the syncytial boundary and changes in thickness of the syncytial boundary. To address the impact of these individual factors on formation and size of nuclear clusters (≥2 connected nuclei), the *in silico* model was run on multiple occasions and this produced well converged statistics. In this model, the number of clusters with more than six nuclei increased over time, mimicking the *in vivo* situation. Figure 5 shows a sample of the post-processed results and the analysis. Mean cluster size increased with nuclear adhesiveness (Fig. 5A). The rate of nuclear movement had a non-linear effect on clustering; initially, increasing the rate of movement increased the rate of nuclear collisions and thus the likelihood of adhesion; however, as the rate increased further, the nuclei were more likely to become unstuck from one another (Fig. 5B). When sites of cytotrophoblast fusion were closer to one another (when *σ* is low), the rate of clustering increased (Fig. 5C). Varying the thickness of the syncytium produced no effect on clustering (Fig. 5D). Examples of the visualisation produced by the *in silico* model when there is high or low nuclear clustering are shown in Fig. 5E.

## Discussion

*In vivo*, SNAs increase in number as pregnancy progresses and are increased in pregnancy complications, most notably pre-eclampsia. This observation suggests that nuclei in the syncytiotrophoblast are not constrained within the cytoplasmic architecture; consequently, we hypothesised that SNA formation is an active process involving cytoskeleton-driven nuclear motility. However, this hypothesis was not borne out; we have demonstrated that there is comparatively little SNA development in term villous explant culture and little nuclear movement within multinuclear syncytia in primary trophoblast culture. Furthermore, neither the stability nor the formation of SNAs is affected by pharmacological disruption of the microfilament or microtubular cytoskeleton. The *in silico* model suggests that formation of nuclear clusters may be promoted by preferential fusion of cytotrophoblast in the region of SNAs or by characteristics of the nuclei (e.g. adhesiveness). This observational data suggest that nuclei in the syncytiotrophoblast are not highly mobile and that it is necessary to seek other mechanisms to explain *in vivo* SNA formation, and why they are increased in pregnancy pathologies.

Both cell and explant models were cultured for extended periods as SNAs are considered to be markers of more mature syncytiotrophoblast, appearing towards term and more often in prolonged pregnancies (Jones & Fox 1978, Al-Allaf et al. 2008). Explants were employed as they maintain the full three-dimensional villous structure and have been previously used to investigate the development of SNAs (Heazell et al. 2008b). However, data obtained in extended static explant culture are necessarily limited and must be treated with caution; the absence of normal endocrine function clearly indicates that syncytial properties are impaired. During culture, the basic structure of the villous tissue remained intact, with minimally increased LDH release and a similar rate of proliferation to that seen *in vivo*. Some evidence suggested that this extended culture exceeds optimum viability of explants and responsiveness after 8 days, including reduced hCG release, loss of E-cadherin and increased M30 staining. However, the persistence of Ki67 staining up to 16 days indicates the potential for fusion of new cytotrophoblasts and formation of new SNAs throughout the culture period. Consequently, the lack of change in SNAs throughout this culture period, which also includes the usual time frame of culture at the beginning, suggests that the lack of SNA formation reflects syncytiotrophoblast behaviour. This evidence opposes judgements that explant vitality decline, due to prolonged culture, is the sole cause for the lack of SNA formation. Both explant and cell models showed signs of apoptosis with increased M30 staining in explants and visible apoptotic cells in primary trophoblast culture. Apoptosis has been linked to SNA formation; however, the relationship between terminal differentiation and apoptosis in the syncytiotrophoblast has not been conclusively established, even if some of the same machinery may be used (Rote et al. 2010, Coleman et al. 2013). The lack of association between increased M30 staining, a terminal product of apoptosis, and SNA formation in placental explants here and elsewhere (Longtine et al. 2012a) provides further evidence that SNA formation is not coincident with apoptosis.

The explant model allows at least a rough evaluation of the potential of SNAs to remain stable, to alter in number or form or to be lost from the tissue during a period of over 2 weeks (admittedly in the absence of maternal circulatory flow). The observations show that there was no evidence of change in explant SNA size or number during a period of over 2 weeks (Fig. 2A and B). In addition to some non-specific delamination (and replacement) of syncytial strips, as previously reported (Siman et al. 2001), particles in the size range of SNAs were shed, which may mimic the release from the placenta during pregnancy as trophoblast deportation (Askelund & Chamley 2011). In tissue, we estimate that there were approximately 3800 SNAs/mg protein and the median number of particles of comparable size to SNAs shed in 48h was 77 per milligram protein, equating to approximately 2% of the total SNAs present (Coleman et al. 2013). Critically, the rate of release did not change over the culture period, suggesting little requirement for replacement during culture. Potentially, particles shed by the placenta into maternal circulation could arise more commonly from syncytial sprouts than SNAs, especially in early pregnancy, although opinion on this is varied in the scientific community (Chamley et al. 2014). Thus, the modest loss and generation of SNAs that we observed in explants could reflect *in vivo* events. These data suggest that the generation of SNAs in the third-trimester placenta is not primarily a developmental device for the disposal of unwanted (and possibly effete) nuclei. It may be that mechanisms other than the release of SNAs, such as loss of syncytial fragments, account for the large quantities of fetal DNA found in maternal circulation (Bianchi 2004).

In primary trophoblast culture, syncytial nuclei were, on average, closer together than nuclei found in adjacent cytotrophoblasts. It is possible that the lower internuclear distances and higher cytoplasmic area per nucleus in syncytiotrophoblast compared with precursor cytotrophoblasts may give the appearance of forming nuclear clusters, rather than an active process by which nuclei are aggregated. The changes in cytoplasmic area may be caused by cytoplasmic redistribution (thinning and spreading) after cytotrophoblast fusion with syncytiotrophoblast but could also be caused by an increase in cell volume. Notably, nuclei within syncytia in cultured trophoblasts moved very little, and the newly formed SNCs (and also SNAs in explant culture) were not vulnerable to separation after inhibitor-based disruption and depolymerisation of the microfilament and the microtubule cytoskeletons.

In explants, the lack of movement could be due to cytochalasin D and nocodazole exerting effects only while present in the culture medium, with normal actin and tubulin structure returning after their removal (Zieve et al. 1980, Friederich et al. 1993, Rhee et al. 2007); however, in primary cytotrophoblasts, there was no treatment-free interval before fixation. If SNAs were actively maintained by actin or tubulin, it was expected that treatment with cytoskeletal disruptors would cause a reduction in SNA size or number. So, despite the pulsatile nature of the treatment in explants, as there was no change in SNA numbers after treatment, these data are most consistent with actin and tubulin function being not required to maintain SNAs. It is also possible that the surrounding cisternae of endoplasmic reticulum may exert a restraining influence. Furthermore, given the low levels of cytotrophoblast proliferation in term placental tissue, it is unlikely that compensatory formation of SNAs could have occurred.

The observations that cytoskeletal inhibitors had no significant effect on SNA or SNC counts suggest there may not be an active nuclear transport mechanism that uses cytoskeletal components present in the syncytiotrophoblast. The lack of nuclear movement despite changes in cytoplasmic area, which would be expected to increase internuclear distance, may indicate that nuclear positioning is mainly determined by the point of initial fusion and then maintained throughout culture. In primary trophoblast, clustering could increase over time as nuclei initially fuse to a similar position, group together and take up a lesser proportion of cytoplasmic area. Meanwhile, expansion of the cytoplasmic area of the syncytium may occur as protein biosynthesis produces secretory machinery, other organelles, membranes and cellular components.

Selected fusion of cytotrophoblasts into the syncytiotrophoblast may be important for SNA formation. Previously, cytoskeletal disruptors have been shown to negatively affect cytotrophoblast fusion if added at 6h (Douglas & King 1993), and this may have had an effect on the early incubation and explant studies. As primary cytotrophoblasts predominantly fuse between 2 and 48h (Kliman et al. 1986), 72h was selected to obtain mature syncytia with SNCs. It is possible that the earlier treatment at 40–42h could have interrupted fusion events (Richard et al. 2009). However, the time between 6 and 24h may have allowed fusion pores to form, so that the cytoskeletal disruption that happened later had a minimal effect on fusion (Richard et al. 2009). The lack of an effect of cytoskeletal inhibitors on cytotrophoblast fusion indicates that most fusion events had already occurred by 40–42h. In explants, it is possible that proliferation of cytotrophoblast and incorporation into the overlying syncytium was inhibited by addition of disruptors at 24h.

Exploration of SNC formation using the *in silico* model, informed by experimental data, identified factors that could be responsible for the formation and maintenance of SNAs/SNCs. These may be grouped into two types of effect: an increased likelihood of collisions between nuclei and an increased likelihood that two adjacent nuclei will become stably associated. For example, increasing nuclear proximity and adherence promotes cluster formation. In contrast, the correlation between the diffusion rate (*D*) of the nuclei and the average cluster size is less clear. As the diffusion rate of the nuclei increases from zero, more collisions occur, resulting in more nuclei forming clusters. However, as this diffusion rate continues to increase, the nuclei do not stay together, and average cluster size decreases. Lastly, if sites where cytotrophoblasts fuse into the syncytium are not uniformly distributed, then the distribution of nuclei is less uniform, with more packed together in certain regions, thereby resulting in more collisions and a higher average cluster size. Our numerical surveys looked at a range of causes of both these effects, which had differing influence on the cluster size distribution. This analysis shows that clustering requires either nuclear diffusion or preferential sites for the introduction of new nuclei into the syncytium, or both. Once regular collisions between nuclei occur, the adherence of the nuclei then plays a significant role in the cluster size distribution. 

Whatever the mechanism by which they approach one another, we have two hypotheses for how nuclei stay together in clusters. First, it is possible that intermediate filaments stabilise nuclear clusters, supported by the observation that grouped nuclei are enmeshed in cytokeratin filament arrays (Jones & Fox 1977, Beham et al. 1988, Bradbury & Ockleford 1990, Coleman et al. 2013). This leads to further speculation that nuclear proximity can activate intermediate filament assembly, possibly relying on the association between elements of the outer nuclear envelope and components of the cytoskeleton. Secondly, proteins on or in the outer nuclear envelope may be able to bind to similarly localised proteins on adjacent intervening cytoplasmic membranes, making nuclei adherent. Although evidence for nuclear adhesion in placenta is limited, proteins on the nuclear envelope or endoplasmic reticulum that are involved with nuclear stabilisation, including elements of the linker of nucleoskeleton and cytoskeleton complex (LINC), SUN and KASH proteins (Starr 2007), are transcribed in the placenta (Supplementary Table 3) (http://www.ncbi.nlm.nih.gov/unigene, 05/10/2015). In yeast and *Caenorhabditis elegans*, the LINC complex participates in transcription, DNA repair and signalling pathways that may be disrupted in SNAs (Kim et al. 2015). Thus, the role of LINC complex merits further investigation in human placenta.

Another possibility is that the position of cytotrophoblast fusion relative to overlying syncytioplasm may contribute to SNA formation, as fusion sites proximal to syncytial nuclei would effectively create groups of nuclei without the need for nuclear motility. Such a mechanism requires either concentrated areas for cytotrophoblast replication or cytotrophoblast motility in tissue; this has not been demonstrated conclusively *in vitro* but remains a possibility. In this context, it is noteworthy that extravillous trophoblasts of the human placenta are dramatically migratory, deeply invading maternal tissues (McKinnon et al. 2001). It will be of interest to determine whether villous cytotrophoblasts are able to move within villous tissue, such movement may be condition dependent, and require the presence of hormones and other factors that are not typically present in *in vitro* culture. This hypothesis deviates fundamentally from the earlier suggestion that nuclei are collected into aggregates by an active process, specifically towards the end of their lifespan in syncytium (Huppertz et al. 2006). The latter hypothesis has been criticised on the basis that transcriptional activity can be found in nuclei within SNAs, that is, they are not simply repositories of inactive, pyknotic nuclei destined for apoptosis. This hypothesis also has relevance for pre-eclampsia, where an increased rate of cytotrophoblast fusion would provide more nuclei that could contribute to SNAs (Arnholdt et al. 1991, Huppertz et al. 2002, Heazell et al. 2006). This formation of SNAs could be further magnified by oxidative stress, as nuclei within syncytial knots particularly show increased levels of oxidative damage (Chaddha et al. 2004, Crocker et al. 2004, Germain et al. 2007, Fogarty et al. 2013). Overall, these hypotheses suggest a mechanism for SNA formation within normal placental development that can be accelerated in ageing and pregnancy complications.

Further experiments are required to further explore the events leading to SNA formation. To study cytotrophoblast fusion and progression into SNAs, floating term placental explants could be denuded of the original syncytiotrophoblast with trypsin and the formation of new SNAs quantified during and after formation of new syncytium. To address the hypothesis that there are preferential sites for fusion, the locations of the newly merged nuclei could be mapped. If newly merged nuclei tend to stay close to each other, this would provide a strong indication that there are preferential fusion sites. If a denuded explant model produced enough syncytiotrophoblast to contain SNAs, it may then provide a starting point for further experiments with inhibitors or BrdU pulse-chase experiments, which could reveal whether newly merged nuclei join with existing SNAs they tend to stay with nuclei of the same metabolic “age”. Further work could also be done to possible interactions between nuclei using explant and cell models. A pull-down assay against nuclear lamins could be performed on syncytiotrophoblast and primary cytotrophoblasts encouraged to fuse in culture. After the pull-down, the contents could be fixed onto slides using a cytospin and imaged to see whether nuclei are always individual or whether SNAs are pulled down, indicative of connections between nuclei. Then, inhibitors or proteases could be added to cultures to see what disrupts nuclear clustering.

In conclusion, nuclei in syncytiotrophoblast appear surprisingly static. SNAs in tissue and SNCs that form in culture are closely enveloped by cytokeratin filaments and neither their formation nor their stability are altered by treatment with actin or tubulin disruptors. Mechanisms other than active nuclear movement within the syncytiotrophoblast cytoplasm are major contributors to SNA formation. Furthermore, our results provide little evidence in support of the hypothesis that SNA “turnover” occurs via specific shedding of SNAs into the maternal circulation. These findings have implications for our understanding of excessive SNA formation in pregnancy disorders. Together our work strongly suggests that further evaluation is warranted into the mechanisms of SNA formation and of their significance in complicated pregnancies.

## Supplementary data

This is linked to the online version of the paper at http://dx.doi.org/10.1530/REP-15-0544.

## Declaration of interest

The authors declare that there is no conflict of interest that could be perceived as prejudicing the impartiality of the research reported.

## Funding

This work was funded by Tommy’s and a Medical Research Council PhD Studentship.

The work performed at the University of Manchester support is appreciated from an Action Research Endowment Fund and the Manchester Biomedical Research Centre.

The work performed at Washington University School of Medicine supporting grants were received from Boehringer Ingelheim Fonds for travel and the NIH to D M Nelson (RO1 HD 29190).
